# Are lower limb biomechanical factors associated with night-time calf cramps in adults? A case-control study

**DOI:** 10.1186/1757-1146-7-S1-A31

**Published:** 2014-04-08

**Authors:** Fiona Hawke, Vivienne Chuter, Joshua Burns

**Affiliations:** 1 Podiatry Program, The University of Newcastle, Ourimbah, NSW, 2258, Australia; 2 Sydney Medical School, The University of Sydney, Westmead, NSW, 2145, Australia; 3 Arthritis and Musculoskeletal Research Group, Faculty of Health Sciences, The University of Sydney / Institute for Neuroscience and Muscle Research / Paediatric Gait Analysis Service of NSW, Sydney Children’s Hospitals Network (Randwick and Westmead), Australia

## Background

Night-time calf muscle cramps are highly prevalent and are associated with reduced quality of sleep and health-related quality of life [1]. The underlying mechanism is poorly understood and no treatment has shown consistent efficacy or safety. The aim of this study was to identify factors associated with night-time calf cramping in adults to explore potential underlying mechanisms and therapeutic targets.

## Methods

160 adults were recruited the Greater Newcastle and Central Coast regions of New South Wales, Australia: 80 who experienced night-time calf cramp at least once per week and 80 age- and sex-matched adults who never experienced lower limb muscle cramping. Participants were assessed using reliable tests of foot/ankle and toe strength, range of ankle dorsiflexion, hamstring flexibility, foot alignment, and calf circumference. Participants also completed a bespoke survey examining health and lifestyle factors, exercise, lower limb symptoms and footwear characteristics.

## Results

Presence of night-time calf cramps was significantly correlated with weakness of foot and ankle inversion, eversion, dorsiflexion and plantarflexion; weakness of toe grip; restricted hamstring flexibility; lower limb tingling sensations; muscle twitching, and coldness of legs or feet in bed at night. Conditional logistic regression identified three factors independently associated with night-time calf cramps: muscle twitching (OR 4.6; 95%CI: 1.6 to 15.5; *p*=0.01), lower limb tingling (OR 4.1; 95%CI: 1.6 to 10.3; *p*=0.003) and foot dorsiflexion weakness (OR 1.02; 95%CI: 1.01 to 1.03; *p*=0.002), which represented other measures of lower limb weakness in the model.

## Conclusion

Night-time calf muscle cramps were associated with markers of neurological dysfunction and potential musculoskeletal therapeutic targets.
